# Microfluidic-Based Technologies for CTC Isolation: A Review of 10 Years of Intense Efforts towards Liquid Biopsy

**DOI:** 10.3390/ijms23041981

**Published:** 2022-02-10

**Authors:** Lucie Descamps, Damien Le Roy, Anne-Laure Deman

**Affiliations:** 1 Univ Lyon, Université Claude Bernard Lyon 1, CNRS, INSA Lyon, Ecole Centrale de Lyon, CPE Lyon, INL, UMR5270, 69622 Villeurbanne, France; lucie.descamps@univ-lyon1.fr; 2 Institut Lumière Matière ILM-UMR 5306, CNRS, Université Lyon 1, 69622 Villeurbanne, France; damien.le-roy@univ-lyon1.fr

**Keywords:** microfluidic devices, circulating tumor cells, liquid biopsy, CTC isolation, downstream analysis

## Abstract

The selection of circulating tumor cells (CTCs) directly from blood as a real-time liquid biopsy has received increasing attention over the past ten years, and further analysis of these cells may greatly aid in both research and clinical applications. CTC analysis could advance understandings of metastatic cascade, tumor evolution, and patient heterogeneity, as well as drug resistance. Until now, the rarity and heterogeneity of CTCs have been technical challenges to their wider use in clinical studies, but microfluidic-based isolation technologies have emerged as promising tools to address these limitations. This review provides a detailed overview of latest and leading microfluidic devices implemented for CTC isolation. In particular, this study details must-have device performances and highlights the tradeoff between recovery and purity. Finally, the review gives a report of CTC potential clinical applications that can be conducted after CTC isolation. Widespread microfluidic devices, which aim to support liquid-biopsy-based applications, will represent a paradigm shift for cancer clinical care in the near future.

## 1. Circulating Tumor Cell Study: Biological Context and Technical Challenges

Cancer is a leading health issue, accounting for nearly 1 in 6 deaths worldwide. By 2040, the disease burden is expected to reach 30.2 million new cancer cases and 16.3 million cancer deaths, according to the Global Cancer Observatory (GCO). Cancer burden can be reduced through early detection and appropriate treatment. In particular, early diagnosis could prevent the formation of metastasis, a multistep process responsible for cancer spread and high morbidity rates [1]. The metastatic process occurs when cancer cells detach from the primary tumor and invade the blood circulation. Then, these circulating tumor cells (CTCs) can extravasate and colonize distant sites, leading to secondary tumor(s). Evidence of this progression through blood circulation was first discovered in 1869 by Thomas Ashworth during an autopsy of a metastatic cancer patient. He observed that cancer cells from a distant site were morphologically consistent with primary tumor cells and concluded that cancer cells were transported through the blood to reach the distant site [2].

In the early stage of the disease, the small size of the primary tumor, as well as the lack of symptoms, are stumbling blocks for early screening. To date, tissue biopsies are core components of cancer patient management to diagnose, assess disease stage, and prescribe appropriate therapeutic regimens. However, biopsies are not only invasive and risky but they may also not fully reflect intratumor heterogeneity; although, the latter has been shown to reduce therapy effectiveness [3]. For all these reasons, tissue biopsies fail to provide a frequent insight into the evolution of tumors.

The study of CTCs may overcome these limitations and become complementary to tissue biopsies. CTCs are released into the bloodstream from primary and metastatic tumors; in addition, besides playing an important role in cancer metastasis [4], they offer a promising clinical potential for cancer diagnosis and prognosis. The isolation of CTCs directly from a blood test, referred to as “liquid biopsy”, has therefore raised strong interest in recent years. These samples can be collected non-invasively and frequently, providing real-time monitoring of tumor evolution and response to treatment. Besides, contrary to a tissue biopsy, which gives a partial “snapshot” of the tumor, liquid biopsy permits investigation of intratumor heterogeneity. Thus, liquid biopsy may lead to changes in the paradigm of cancer diagnosis and management by providing earlier diagnosis and more personalized treatment [5]. 

Nevertheless, until now, isolating CTCs has been a technical challenge, limiting their wider use in research and clinical studies. The main issue is the rarity of CTCs (1–1000 CTCs per mL) among a high background of blood cells (10^9^ red blood cells (RBCs) and 10^7^ white blood cells (WBCs) per mL). Moreover, their isolation is challenging, due to th following characteristics: (i) a morphology similarity with some WBCs, reducing size-based sorting effectiveness; (ii) a phenotypic heterogeneity, which makes the use of biomarkers more complex and limits the effectiveness of biomarker-based separation. The latter challenge results from the epithelial-to-mesenchymal transition (EMT) that CTCs can undergo [6], which results in a decreased expression of epithelial markers and the appearance of a mesenchymal phenotype, which is associated with an increase in the capacity of invasiveness, immune escape, and metastasis [7]. Finally, the isolation of CTCs should be achieved while preserving their integrity for downstream characterizations. 

Until now, Cellsearch™ (Veridex, Raritan, NJ, USA) is the only device approved by the US Food and Drug Administration (FDA) for CTC detection and enumeration for clinical use. The device uses ferrofluid particles, coated with antibodies, targeting the epithelial cell adhesion molecule (EpCAM) for the enrichment of CTCs from the patient’s blood. Isolated cells are subsequently immunostained with fluorescently labeled antibodies and then counted using automated cell image capture and analysis. Since its introduction in 2004, the CellSearch™ system has been used as a diagnostic and prognostic tool in patients with metastatic breast, colorectal, and prostate cancer [8]. Although this system is considered a “gold standard” for CTC detection, it has several drawbacks. The detection sensitivity of this approach highly depends on epithelial markers, which results in a low recovery for cells that underwent EMT. CellSearch demonstrates a recovery rate of only 2% for mesenchymal breast cancer cell lines [9]. In addition, the system enriches CTCs with a high background of contaminating WBCs, leading to low purity (0.01–0.1%) [10] and limiting further analyses. As a negative enrichment approach, the RosetteSep™ technology (STEMCELL Technologies, Vancouver, BC, Canada) is based on bispecific antibodies that can crosslink RBCs and WBCs to form clusters (cell rosettes), which can be subsequently removed through density gradient centrifugation [11]. However, besides requiring different kits depending on the cancer type, the isolation of CTCs from the plasma and density gradient interface is extremely challenging and may further compromise their biological integrity. Another approach based on their size, the ISET (isolation by size of epithelial tumor cells, RareCells, France) kit is used to isolate CTCs [12]; however, similarly to CellSearch™, this method only monitors epithelial cells. Thus, there is still an unmet need for specific and sensitive isolation of clinically relevant CTCs, required for their further characterization. 

## 2. Microfluidic Devices—New Prospects in CTC Isolation

**Figure 1:**
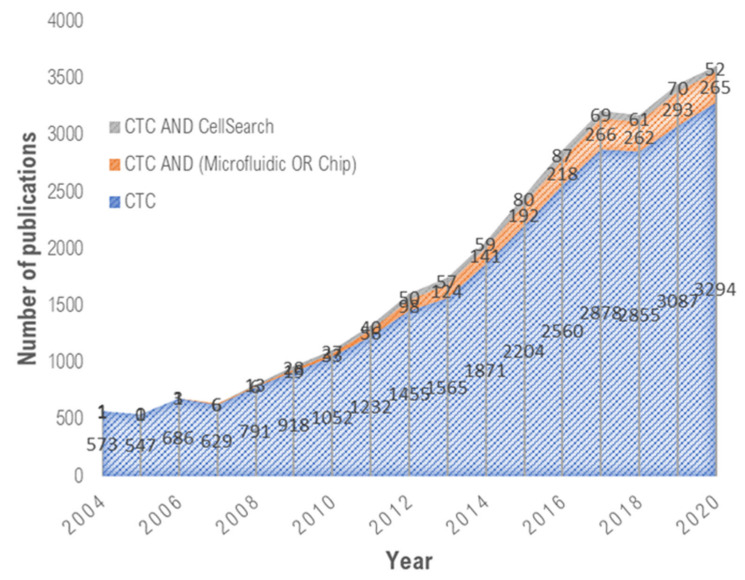
Emerging microfluidic technologies for CTC isolation. Data collected from Web of Science advanced search using specific keywords (“CTC”, “CellSearch”, “Microfluidic”, “Chip”).

Over the past decade, microfluidic devices have emerged as promising tools to address these challenges (Figure 1). Microfluidic devices possess unique advantages over conventional approaches, among which one can cite the following: (i) their micrometric dimensions and laminar flow nature, enabling precise object manipulation and single-cell study; (ii) the handling of small volumes, which facilitates the analysis of rare or expensive samples and speeds up the processes, leading to cost-effective devices; (iii) the integration of various functions (mixing, focusing, sorting, trapping, detection, etc.) into a single device, leading to compact and portable systems, and therefore opening the way for the implementation of point-of-care devices [13,14]. All these merits demonstrate that microfluidic devices offer new prospects in CTC study.

Several microfluidic technologies awaiting FDA clearance have been commercialized for CTC isolation, such as Parsortix^®^ (ANGLE plc, Surrey, UK) [15], ClearCell^®^ FX1 (Biolidics Limited, Mapex, Singapore) [16], and VTX-1 (Vortex Biosciences, Menlo Park, CA, USA) [17]. These technologies are based on the difference in CTC size and deformability compared with blood cells, but other physical properties, such as density and electrical charges, can also be studied. Other microfluidic-based isolation technologies rely on the biological properties of CTCs and exploit their specific surface marker expression. 

In the following sections, relevant microfluidic-based CTC isolation devices will be reported, categorized by the separation method adopted (physical- or biological-based), which will be compared by examining the figures of merit, detailed in Box 1. The ideal device would have high recovery and purity, high throughput for sample processing, and the ability to collect heterogeneous and viable CTCs for downstream analysis to provide clinical information. Recent microfluidic devices for CTC isolation are mainly reported in this review, and several reviews can be studied to retrace the research efforts in this field [5,18,19,20,21,22,23,24].

Box 1 **Figures-of-merit data for microfluidic-based CTC isolation devices.**

**Recovery:** The fraction of injected CTCs that are collected downstream.

**Purity:** The fraction of CTCs among the collected cells in the output. Sample purity determines the panel of downstream analyses that can be performed.

**Throughput:** The applied flow rate for blood sample processing.

**Viability:** The assay’s ability to preserve the viability of recovered CTCs.

**Clinical yield or clinical recovery:** The number of isolated CTCs obtained from patient samples with a defined cancer type.

**Clinical sensitivity:** The minimum number of detected CTCs in patient samples.

**Clinical relevance:** The biological and therapeutic information that can be obtained beyond the enumeration of the isolated CTCs.

## 3. CTC Isolation Using Physical-Based Separation Technologies

**Figure 2:**
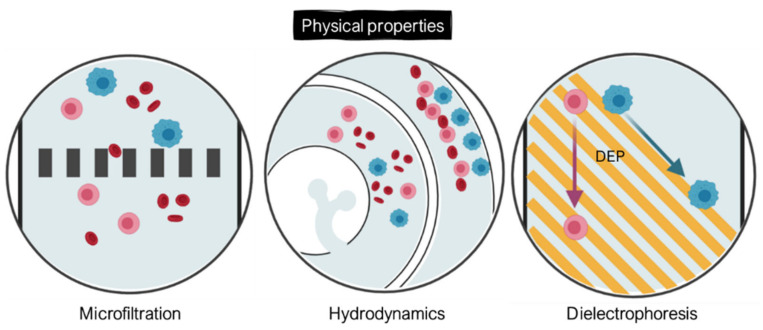
CTC-enrichment technologies based on their physical properties through integrated microposts (microfiltration), specific microchannel designs (hydrodynamics), or application of electric fields (dielectrophoresis). CTCs appear in blue, while RBCs and WBCs are represented in red and pink, respectively. Created with BioRender.com (Accessed on 31 January 2022).

Numerous studies (morphological, mechanical, electrical) have highlighted the physical and biomechanical properties of CTCs, enabling their distinction from other blood cells [25]. Indeed, most of CTCs have a bigger size (17–52 µm) than RBCs (6–8 µm) and WBCs (7–15 µm for the majority, 20 µm for monocytes), a higher nuclear–cytoplasmic ratio, and an intricately folded membrane. Moreover, their mechanical properties allow them to deform when passing through blood vessels [26]. This low stiffness of the cytoplasm plays a part in the metastatic process: it facilitates CTC migration towards secondary sites and increases their resistance to the shear stress involved in the vascular system [27]. Finally, cytoskeletal remodeling has an impact on membrane structure conservation, which leads to a modification of the electrical surface charges and, therefore, of the electrical properties of CTCs. This is why separation methods based on physical criterion (size, deformability, electrical properties) were developed to isolate CTCs from blood. Some of the most widespread separation techniques based on CTC physical properties are illustrated in Figure 2.

### 3.1. Microfiltration Separation Methods

Microfiltration techniques have been implemented in microfluidics for CTC isolation [10,11,12,13,14,15,16,17,18,19,20,21,22,23,24,25] and consist of flowing a blood sample through micrometric constrictions to capture CTCs, while other blood cells pass through. This separation method relies on cell size properties, as well as on a combination of size and deformability criteria. Several strategies were studied for CTC isolation: microstructure post filters [28,29,30,31,32], microporous membranes [33,34,35,36,37], or microfluidic constrictions [38,39,40,41,42,43]. 

The first approach consists in integrating an array of micrometric-sized posts that act as filters to capture CTCs. To this end, several microstructure post shapes were imagined: from microellipse [30] to micropillars [31,32] and funnel constrictions [29]. Chen et al. integrated an array of microellipse filters which consists of microfluidic slits in gradually narrowing series [30]. Ellipse-shaped filters reduce friction and shear stress, therefore preserving tumor cell viability. They achieved a capture efficiency of 90% of cancer cells at 1 mL/h with a viability of 96%. Then, they processed blood samples from four metastatic breast cancer patients and nine non-small-cell lung cancer (NSCLC) patients to evaluate microellipse clinical performances. CTCs were detected positive for the 2–3 mL blood samples of all the patients, with 4 patients having more than 20 CTCs. Nevertheless, additional concerns include the low sample capacity resulting from filter clogging with cells, as the usual blood volume processed in clinical experiments is 7.5 mL. In addition, in this developed device, CTCs cannot be recovered to conduct downstream analysis. 

**Figure 3:**
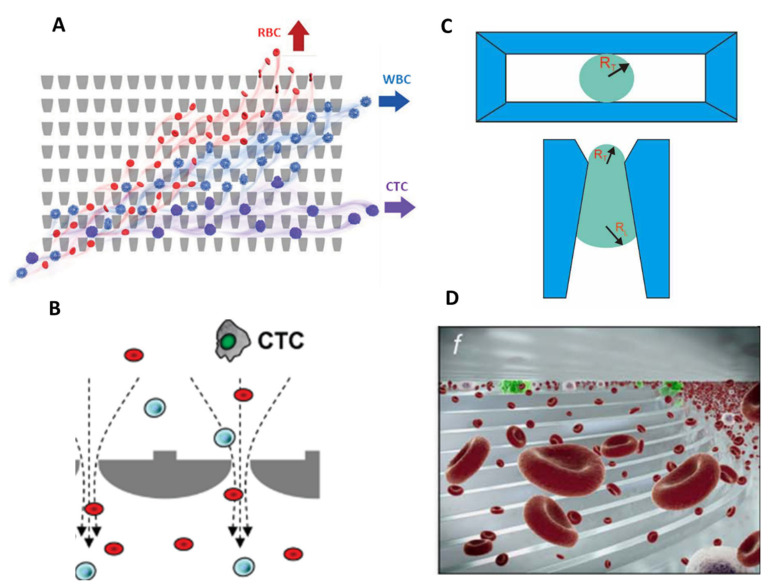
Microfiltration separation technologies. (**A**) Microfluidic ratchets for continuous CTC separation. Whole blood is infused from the bottom-left corner of the funnel array and cells travel in a zigzag diagonal path until they reach a blocking funnel row, where they proceed horizontally toward the outlet reservoirs. The size of funnel constrictions is gradually reduced from the bottom row to the top row within the 2D array. Reprinted from [29] with permission (http://creativecommons.org/licenses/by/4.0/) (Accessed on 26 October 2021). (**B**) Circular microcavity array (MCA) filter. The size of the microcavities was optimized in order to trap CTCs on the microcavities while letting blood cells flow through the filter. Reprinted with permission from [44]. Copyright 2010 American Chemical Society. (**C**) Pyramidal MCA filter. Top view and vertical section of cell retention in a pyramidal MCA. R_T_ and R_L_ are, respectively, the radius of the curvature of the trailing and leading edges of the cell. Reprinted from [34], copyright 2019, with permission from Elsevier. (**D**) The Parsortix™ system. Blood is forced along a series of channels with a cross-sectional gap that gradually decreases the dimension of the fluid path and retains CTCs based on their deformable nature and size. Reprinted with permission from [41] under the Creative Commons CC-BY-NC-ND license.

Strategies were investigated by other researchers to tackle these issues. Park et al. developed a deformability-based device to enrich viable CTCs directly from whole blood by integrating funnel-shaped constrictions with openings smaller than the diameter of the cell [29]. The device takes advantage of the microfluidic ratchet mechanism, which relies on the distinct deformability (or more precisely, squeezability) of CTCs, relative to hematological cells. They fabricated a 2D array of funnel constrictions, where the size of the funnel opening is gradually reduced (from 18 to 2 µm). Using continuous oscillatory flow, whole blood is infused from the bottom-left corner of the funnel array and cells proceed to travel in a zigzag diagonal path until reaching a blocking funnel row, where they proceed horizontally toward the outlet reservoirs (Figure 3A). The combination of oscillatory flow and asymmetrical deformation enables the whole blood to be processed continuously and eliminates clogging issues. They achieved a capture efficiency between 77% and 90% in spike samples, with an enrichment factor varying from 5000 to 14,000. The device was validated with 20 clinical samples from patients with metastatic prostate cancer and results were compared with the CellSearch™ system. The microfluidic ratchets presented a high sensitivity and allowed the detection of a median 178 CTCs/7.5 mL compared with a median 7 CTCs/7.5 mL with the CellSearch™ system. CTC counts were obtained from 2 mL of patient blood using the microfluidic ratchet device, and were, therefore, scaled to 7.5 mL, to be compared with results obtained using the CellSearch system. As the throughput of microfluidic ratchets is relatively low, about 1 mL/h, 2 devices were run in parallel to process the 2 mL of blood (1 mL/device). However, this device has not yet been able to process 7.5 mL of blood (standard volume for protocols), which could increase the probability of recovering CTCs.

The second filtration approach consists of microporous membranes, which leads to higher throughput (>3 mL/h). Hosokawa et al. developed a microfluidic device equipped with a nickel-based microcavity array (MCA) filter to enrich CTCs from blood samples (Figure 3B). The first fabricated device consisted of 10,000 circular cavities, with diameters of 8–11 µm, and a distance of 60 µm between each of them [44]. The MCA filter was sandwiched between an upper substrate, which consists of a microchamber, sample inlet, and an outlet; additionally, there is a vacuum line in the lower substrate, to produce negative pressure and enable cell entrapment. The device showed very high capture efficiency in non-small-cell lung cancer (NSCLC) cells, with a separation efficiency as high as 97% in 1 mL of blood spiked with 10–100 cells, processed within 15 min. However, once single cells are trapped on the circular microcavities, other cells are driven towards unoccupied microcavities and pass through under higher pressure. This excessive flow resistance causes cell deformation and leads to the escape of small tumor cells such as small-cell lung cancer (SCLC) cells from the circular microcavities, therefore reducing the capture efficiency. The researchers further optimized the structure of the MCA to successfully separate small-sized tumor cells, like those found in SCLC. They fabricated rectangular-shaped microcavities, with a width of 5–9 µm and a length of 30 µm [45]. With this optimized rectangular MCA, they reached higher recovery and purity rates than those obtained with the circular MCA for small tumor cells. They conducted a clinical study on a newly automated MCA system and demonstrated the superiority of the system in comparison with CellSearch™ for the detection of CTCs in patients with NSCLC [36]. Nevertheless, for patients with SCLC, the CellSearch™ system showed better performances. This can be explained by the dependence of the MCA system on the difference in cell size between tumor cells and normal blood cells, which inevitably results in a loss for tumor cells of smaller size such as SCLC cells. Further development should therefore be made to achieve better sensitivity. 

Recently, another MCA structure was imagined by Yin et al., since vertical entrances (presented above) keep blood cells from entering and escaping the microcavities and thus decrease the efficiency and purity of separation [46]. They integrated an MCA filter with pyramidal microstructures into a microfluidic device for CTC enrichment from raw blood samples [34] (Figure 3C). The silicon-based microcavity array was fabricated by lithography and induced coupled plasma-reactive ion etching (ICPRIE) technologies to obtain 10,000 microcavities in a 14 × 14 mm filter. Microcavities have a length of 30 µm and a width of 8 µm and are spaced 60 µm from each other. The device is fabricated by adhering the top and bottom polydimethylsiloxane (PDMS) layers on the pyramidal MCA. In this device, the slope at the entrance of the cavity, combined with a gradual increase in the channel size from top to bottom, facilitates the deformation and escape of blood cells. Approximately 80% of spiked tumor cells were separated from 1 mL of whole blood at a flow rate of 6 mL/h, and less than 0.003% of unwanted WBCs were captured. Furthermore, the microfluidic chip successfully identified CTCs in 5 out of 6 blood samples from breast or lung cancer patients, with a range of 5–86 CTCs per mL. Further clinical sample processing should be performed to assess the clinical readiness of the device. However, due to the heterogeneity of CTCs, smaller CTCs could not be captured by microcavities. The same team later used functionalized microspheres to increase the size of CTCs and to enable better discrimination against WBCs [47], but in doing so, they undid the benefit of the label-free separation method.

Finally, the last filtration approach consists of narrowing the dimensions of the fluidic path, through which cells flow to entrap CTCs. Hvichia et al., developed a semi-automated separation system, the Parsortix™ (Figure 3D), which is currently awaiting FDA clearance for its use in metastatic breast cancer patients. The system achieved an average capture efficiency of 64% at high throughput (10 mL/h), with high purity (200–6000 leukocytes left) and high viability (99%) [41]. The performance of the system was compared to CellSearch™ in 26 clinical samples. The ranges of CTC counts were 0–6.5/mL for Parsortix™ and 0–33/mL for CellSearch™, respectively. Despite a lower sensitivity, the major advantage of Parsortix™ is the recovery of viable tumor cells with which to perform molecular and functional downstream analysis. For personalized treatment, it is also crucial to understand the biological processes coming into play in drug susceptibilities, which can be established by proteomic profiling of CTCs. Recently, Armbrecht et al. developed a microfluidic device integrating a bead-based assay for the direct quantification of proteins secreted by both single CTCs and CTC clusters [38]. Size-based filtration is a quite straightforward way to isolate CTC clusters since even two-cell clusters are sufficiently larger compared to WBCs to allow a clean separation [48]. These clusters, although rare, are the most aggressive subset of CTCs and could affect clinical decisions [49,50]. The device consists of two layers, the top one containing a channel network with trapping units, and the bottom one containing pneumatic, donut-shaped valves. The trapping units, 1152 in total, are arranged in 4 parallel segments to reduce processing times of 6.5 mL whole blood samples. The integrated device could achieve capture, isolation, and subsequent analysis within a single trapping unit. CTCs and CTC clusters are first captured through a reduction in channel heights from 25 to 7.5 µm and retained by 2 micropillars forming a 2D constriction. CTCs are then co-captured with beads and the valves are actuated to form the analysis chamber in which a sandwich immunoassay will be performed. They achieved capture efficiencies superior to 95% for various cell lines at a flow rate of approximatively 1 mL/h, with <5% of co-captured WBCs. Using this system, the secretion level of granulocyte colony stimulating factor (G-CSF), which indicates acute inflammation [51], was directly quantified. The device enabled the processing of 6.5 mL untreated blood samples within 5–6 h. Further improvement could be made to reduce the processing time, as well as to achieve a fully automated protocol.

### 3.2. Hydrodynamic Separation Methods

Further label-free separation methods using hydrodynamic forces were developed. Compared with microfiltration techniques, hydrodynamic isolation exerts low fluidic stress on cancer cells as they do not pass through physical obstacles. Besides, samples are processed at high flow rates to ensure the generation of relevant hydrodynamic forces, which leads to high-throughput sorting, while cancer cells can be retrieved for subsequent analysis. Some of the most promising hydrodynamic isolation strategies can be classified into size-dependent deterministic flow pathways in pillar arrays (so-called deterministic lateral displacement), inertial migration of cells in a multi-flow straight microchannel, inertial focusing in spiral microfluidic channels (so-called dean flow fractionation), and microfluidic vortices generated in micro-reservoirs aside the channel.

#### 3.2.1. Deterministic Lateral Displacement

**Figure 4:**
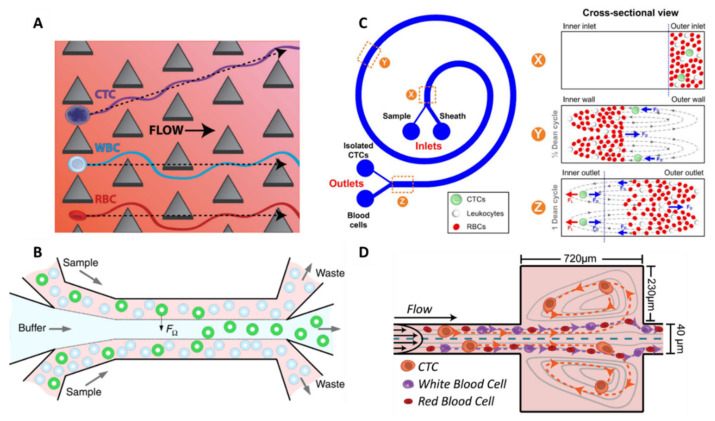
Hydrodynamic separation technologies. (**A**) Deterministic lateral displacement. An array of triangular posts with a gap distance of 42 µm was integrated into a microfluidic channel for continuous CTC sorting. CTCs constantly collide with posts and are forced to move laterally following the post arrangement. Reprinted with permission from [54] under a Creative Commons Attribution (CC BY) license. (**B**) Inertial focusing in a straight channel. The multi-flow configuration leads to the lateral migration of CTCs from the sample streams into the buffer stream due to the predominancy of the rotation-induced lift force (FΩ). Reprinted with permission from [58] (http://creativecommons.org/licenses/by/4.0/) (Accessed on 26 October2021). (**C**) Dean flow fractionation. CTCs move toward the inner wall of the spiral microchannel, because of the balance of inertial lift force and Dean drag force, while small cells (RBCs and WBCs) flow toward the outer wall. Reprinted with permission from [59] (http://creativecommons.org/licenses/by-nc-nd/3.0/) (Accessed on 26 October 2021). (**D**) Microvortices. Cells flowing through a series of expansion–contraction reservoirs experience multiple microvortices because of the shear gradient lift force in expansion reservoirs. CTCs are collected in the center of the vortices. Reprinted with permission from [60] (http://creativecommons.org/licenses/by/4.0/) (Accessed on 26 October 2021).

The deterministic lateral displacement (DLD) utilizes an array of posts within microchannels, where each post is laterally shifted at a set distance from the previous post. By optimizing the gap distance and post size, one can determine a critical size. Cells smaller than the critical size flow between the post gaps, while larger cells constantly collide with posts and are forced to move laterally following the post arrangement, achieving continuous CTC sorting. When DLD was firstly reported, a circular shape of post was used, with a gap of 10 µm [52,53]. However, isolation of CTCs from a cancer patient’s bloods by the DLD method easily results in clogging. Thus, Loutherback et al. replaced circular posts with triangular ones and increased the gap distance to 42 μm (Figure 4A). They achieved >85% capture efficiency of spiked breast cancer cell lines from whole blood at high flow rate (600 mL/h) [54]. DLD, combined with filter structures, has also been reported by Liu et al., leading to high separation efficiency (>96%) with high purity [55]. Recently, Au et al. fabricated a two-stage DLD device to isolate intact CTC clusters [56]. The first stage is a “standard” DLD step, designed as an array of 50 µm diameter micropillars with 63 µm gaps between each one to extract large clusters (>30 µm) from whole blood without clogging. Remaining clusters, cancer cells, and blood are shuttled into the inlet of the second device stage, which uses asymmetrical pillars and height restrictions to extract smaller clusters based on the inherent asymmetric nature of multicellular aggregates. The novelty of this 2-stage capture strategy rests in its enrichment of small and large clusters (100 cells) in 2 distinct outputs. These size-enriched outputs may be useful to further investigate the influence of cluster size on the function, composition, and potency of clusters. In comparison with small clusters, large clusters appear to harbor heterogenous cells (e.g., fibroblasts, endothelial, or tumor-infiltrated myeloid cells), increasing tumor cells viability within CTC clusters, and facilitating metastases formation [57].

#### 3.2.2. Inertial Focusing

Another hydrodynamic size separation technology utilizes inertial focusing to create size-dependent equilibrium positions within the channel. This phenomenon relies on the balance of lift forces arising from the curvature of the velocity profile (the shear gradient lift) and the interaction between the cell and the channel wall (the wall effect lift) for Reynold number of the order of 1 or greater [61]. It results in a lateral ordering of cells according to their size: larger cells migrate to the channel centerline. Zhou et al. implemented a inertial-based separation in a simple straight channel for CTC isolation from untreated whole blood in the first developed device [62], or from RBC-lysed blood in their more recent device [58]. Indeed, the considerable contamination from RBCs compromises its outcome. In this case, cell migration is dictated by the rotation-induced lift force, which is the predominant inertial force. The device is designed as a multi-flow configuration in a straight microchannel, 150 µm in width and 50 µm in height, with 2 inputs and 2 outputs (Figure 4B). Buffer (PBS) and sample are injected at the inner inlet and outer inlet, respectively, forming three flow streams in the main channel. The buffer flow is sandwiched between the two sample flows in the middle of the channel, and, under the influence of inertial forces, larger target cells migrate laterally away from the sample streams into the buffer stream. The authors set 15 µm as the cut-off size to differentiate CTCs from WBCs, which is obtained for a channel length of 20 mm, according to previous work estimations [63]. With this cut-off size, this device is not suitable for the recovery of CTCs smaller than 15 μm. The performances of the device were first studied using spiked cancer cells at clinically relevant concentrations (10 cells per 5 mL and above) and a recovery rate superior to 93% was achieved, with high purity (>87%). The clinical potential of the device was also demonstrated after successful CTC detection from 6 out of 8 NSCLC patients. Further applications were conducted with this device, including CTC cluster isolation and molecular characterization [64], as well as on-chip cell culture [65].

Other channel geometries are reported in inertial microfluidics for CTC separation and can generate secondary flows that create additional hydrodynamic effects beyond shear gradient and wall effect lift forces for improved separation. In a spiral microchannel, the channel curvature introduces two symmetrical counter-rotating flows, called Dean vortices, within the transverse plane of the channel. This Dean flow fractionation (DFF) separation method causes large cells (CTCs) to move toward the inner wall, because of the balance of inertial lift force and Dean drag force, while small cells (RBCs and WBCs) flow toward the outer wall. Cells with different size can thus be collected in two separate outlets. Spiral microchannels can provide inertial focusing but in a much smaller footprint [59]. The group of Lim has carried out a lot of work on the DFF isolation approach for CTC enrichment in recent years [66,67,68,69,70]. In 2013, they reported a single spiral microfluidics and achieved a cell line recovery rate of 85% at a flow rate of 3 mL/h [66] (Figure 4C). Clinical validation was demonstrated with 100% sensitivity in samples from patients with metastatic lung cancer with a purity of 0.1–10%. They further improved the separation throughput to 12 mL/h while preserving purity by fabricating a multiplexed spiral chip which consists of a three-stack spiral chip and including an RBC lysis step [69]. This RBC lysis pretreatment step substantially removes blood contaminants and reduces the overall cell concentration, therefore limiting the undesired cell dispersion due to cell–cell interaction [70]. The clinical use of this new chip was demonstrated by detecting CTCs from 100% (10/10) of blood samples collected from patients with advanced-stage metastatic breast and lung cancers. With this device, between ~10s and 10,000s of WBCs per ml of blood (median from 30 samples = 3109 WBCs per ml) remain after spiral chip processing; this purity was sufficient for downstream sequencing or fluorescence in situ hybridization (FISH) analysis [67]. They had to compromise for either high CTC recovery or high WBC removal. This separation technique has been commercialized as ClearCell FX1^®^ (Biolidics) and has recently been recognized through its ISO certifications (Europe: CE-marked for In Vitro Diagnostic, US FDA and China NMPA (National Medical Products Administration) Class I Medical Device registered). 

Recently, Lin et al. created the Labyrinth device to address the challenge of focusing on smaller cells, such as WBCs, which remain unfocused in most DFF technologies. It was achieved by incorporating numerous sharp corners placed across the flow pattern to enhance Dean forces for the migration of smaller cells to their equilibrium positions [71]. It resulted in a high recovery rate of >90% with cell lines from breast, pancreatic, prostate, and lung cancers, with high purity (600 WBCs/mL) and at an extremely high flow rate of 150 mL/h. The combination of long loops and sharp corners leads to separated focusing of both large (CTCs) and small (WBCs) cells, while most spiral devices have to compromise for either low CTC recovery or low WBC removal. The device was clinically validated in pancreatic and breast cancer samples with a sensitivity of 95% (72 out of 76).

#### 3.2.3. Microvortices

Finally, similarly to DFF, another inertial-based device was reported using contraction–expansion arrays, whose cross-sections periodically widen and narrow to differentiate the focusing positions of particles depending on their sizes. When cells flow through a series of expansion–contraction reservoirs within a microchannel, they experience multiple microvortices because of the shear gradient lift force in expansion reservoirs. Cells larger than a critical size are collected in the center of the vortices; therefore, CTCs can be separated from other blood cells using this method. The Vortex technology was developed by the group of Di Carlo and has been well described for CTC enrichment over the years [60,72,73,74] (Figure 4D). Sollier et al., first developed and optimized the Vortex chip by varying several parameters such as channel dimensions and flow rates to achieve maximum trapping efficiency and purity [72]. Trapped CTCs in the vortices are released on-demand by lowering the washing buffer (PBS) flow rate. High blood volumes (10 mL volume samples of 20× diluted blood) were processed at high throughput (22.5 mL/h) and spiked cancer cells were concentrated to a small final volume of 300 µL. They obtained a capture efficiency of 21% and a purity as high as 89%. They further optimized the platform into an advanced Vortex HT chip by replacing the long, straight, upstream focusing channel with serial 1000 µm-spaced reservoirs to improve cell capture and increasing parallelization from 8 to 16 channels. They achieved improved capture efficiency (up to 83%), high purity (28.8 ± 23.6 WBCs/mL), and ultra-high throughput (480 mL/h of whole blood) [60]. The Vortex HT chip enabled the coupling of in-flow, label-free cell enumeration on bright-field images with various standard assays downstream, such as cytology and cytogenetics [73]. They assessed the feasibility of characterizing the anaplastic lymphoma receptor tyrosine kinase (ALK) gene rearrangement by FISH in CTCs isolated from patients with NSCLC. Recent studies have demonstrated that detecting ALK rearrangements can be of clinical value for physicians to select more effective therapies [75]. Finally, they highlighted the phenotypical heterogeneity of CTCs from 22 patients with advanced prostate cancers [74]. A fraction of the collected cells (10.4%) did not express epithelial prostate, markers while some instead expressed markers of epithelial–mesenchymal transition. This Vortex technology has been commercialized as the VTX-1 Liquid Biopsy System by Vortex Biosciences.

Inertial-based sorting methods therefore have numerous advantages: high throughput, high recovery, and CTC retrieval for subsequent analysis. However, the main drawback of this method is the risk of CTC loss during the process, leading to a false negative result. Reducing the cut-off size can help to minimize this loss with the tradeoff of the increased contamination of white blood cells, reducing purity.

Hence, despite the straightforward and label-free separation methods based on CTC size, the non-specificity of the size criteria limits their efficacies. Indeed, the separation methods were optimized on cancer cell lines, but studies have shown the morphological heterogeneity of CTCs found in patient bloods, from round to oval shapes, and with diameters varying from 4 to 30 µm [76]. Besides, some WBCs have shown diameters as big as those of CTCs, leading to low purity. For all these reasons, other physical-based strategies were investigated to perform CTC isolation. 

### 3.3. Dielectrophoretic Separation Methods

Besides size-based and deformability-based separation methods, dielectrophoresis (DEP) utilizes the electrophysical properties of CTCs to isolate them under a nonuniform electric field. When applying an AC voltage across two electrodes of different sizes, the non-uniform distribution of the charges generates a net DEP force will move the cell either towards the higher electric field gradient region (so called “positive DEP”), or in the opposite direction, towards the lower electric field gradient region (negative DEP). At a given electric field frequency (so-called crossover frequency) and depending on the electrical conductivity of the cells and its suspending medium, cell may experience either positive DEP (higher cell conductivity) or negative DEP (higher medium conductivity). Generally, viable cells express negative DEP at low frequencies and positive DEP at high frequencies. In particular, cells with different membrane surface area exhibit distinct DEP frequency responses. As previously mentioned, CTCs are larger, but they also present a 60% greater surface area than a WBC of the same size [19], which gives them larger capacitance per unit area and enables their controlled motion in a DEP-based device. 

**Figure 5:**
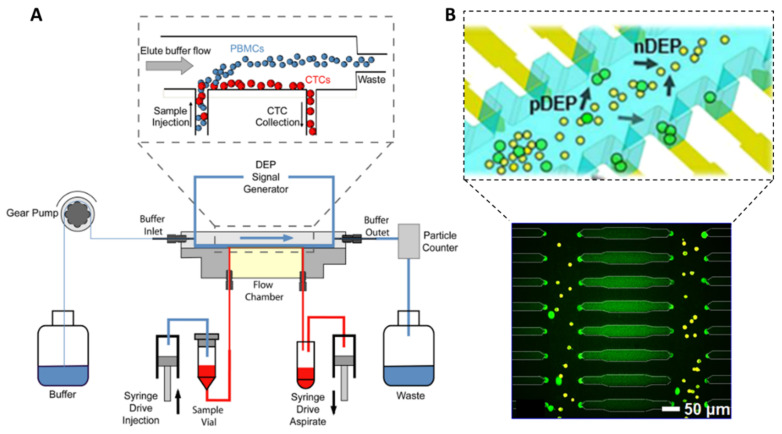
Dielectrophoretic separation technologies. (**A**) The ApoStream™ system. The flow chamber applies an AC electric field to the sample at a frequency in between the crossover frequency of CTCs and WBCs to pull the former towards the chamber floor (positive DEP) and repel the latter (negative DEP). Reprinted from [78] with the permission of AIP Publishing. (**B**) Wireless bipolar electrode (BPE) array. Capacitive charging of the electrical double layer at the BPE tips transmits an AC field across the device and provides sufficient electric field gradients to exert DEP trapping force. Cancer cells (in green) experience positive DEP and are trapped at the electric field maxima around the BPE tips (single-cell capture), while other cells (in yellow) undergo negative DEP and remain in fluid flow. Reprinted (adapted) with permission from [84]. Copyright 2017 American Chemical Society.

Two DEP-based devices were commercialized ten years ago for CTC isolation, the DEPArray™ (Menarini Silicon Biosystems Spa, Bologna, Italy) for single CTC DEP trapping [77], and the ApoStream™ (Precision for Medicine, Inc., Houston, TX, USA) for continuous CTC enrichment [78]. The DEPArray™ device consists of an array of individually controllable microelectrodes which—when the electric field created above a subset of electrodes is in counter-phase with the electric field of adjacent electrodes—generate up to tens of thousands “DEP cages”. Each DEP cage is able to capture a CTC in stable levitation, avoiding contacts between the cells and the surface. DEPArray™ is frequently used as a downstream single-CTC isolation technique using the recovered CTC samples from CellSearch ™ system to perform subsequent molecular characterizations [79]. The Apostream™ system integrates interdigitated electrodes located on the floor of the chamber, above which cells are flowing (Figure 5A). The sample is injected at a low flow rate into the bottom of the flow chamber to minimize cell levitation and to ensure cells stay within the effective DEP field [80]. By applying an AC voltage signal at a frequency in between the crossover frequency of cancer cells and WBCs, cancer cells are attracted by positive DEP forces towards the electrode plane and collected in the bottom collection output, while WBCs are repelled by negative DEP and levitate towards the top waste output. They achieved a >70% recovery efficiency for both epithelial and mesenchymal cell lines, with a purity of approximatively 0.3% (~10,000 WBCs/mL) at a flow rate of 1 mL/h. The device has been successfully employed in clinical samples for the isolation of CTCs from epithelial and non-epithelial cancer types [81,82,83].

Other DEP strategies were reported for CTC isolation, including DEP field flow fractionation (DEP-FFF) and optically induced dielectrophoresis (ODEP), but they suffer from relatively low throughput in the range of 0.01−1.0 mL/h [85,86,87]. Recently, Li et al., fabricated arrays of wireless bipolar electrodes (BPE) generating an AC field across channel walls and attracting CTCs towards micropockets located along the microchannel walls [84]. These micropockets aligned to the BPE tips provide discrete capture sites with defined volume, thus enabling single-cell capture (Figure 5B). They showed that over 80% of pockets captured individual MDA cells at a flow rate of 0.1 mL/h. In addition, they demonstrated the processing of 7.5 mL standard blood volume within their parallel-channel device and removed the need for wires. Further developments are investigated to improve the throughput, by increasing the device footprint and reducing DEP buffer volume. 

Thus, DEP-based separation methods provide high recovery rates, but their implementation can be challenging. Indeed, they require specific electrode geometries and controlled microfabrication. In addition, DEP separation systems rely on cells polarization differences; therefore, any cell exhibiting a damaged membrane may influence isolation efficiencies. The use of specific buffers, such as sucrose, can induce osmotic stress and cause leakage of cytosolic ions over time [19]. Finally, the high conductivity of blood can modify the separation performances—therefore limiting efficacy and purity.

### 3.4. Summary of Physical-Based Separation Methods

Some advantages and limitations of each presented separation method can be found in Table 1. Hydrodynamic separation methods offer higher throughput and a more robust implementation compared with microfiltration and dielectrophoretic sorting techniques. The performances of reported technologies for cell line studies and clinical studies are then summarized Table 2 and Table 3, respectively.

More recently, acoustophoresis has evolved as a promising field for CTC sorting [88,89,90,91,92]. This method relies on the migration of cells under the influence of acoustic waves, according to their size, density, and deformability. Acoustic-based approaches offer a contactless, simple, cost-effective, and versatile separation. More importantly, this method could overcome the potential jeopardization of cancer cell integrity due to high flow rate requirements of the inertial separation or the low conductive medium of DEP. Thus, acoustophoresis may offer interesting perspectives for the separation of CTCs based on their physical properties.

## 4. CTC Isolation Using Biological-Based Separation Technologies

**Figure 6:**
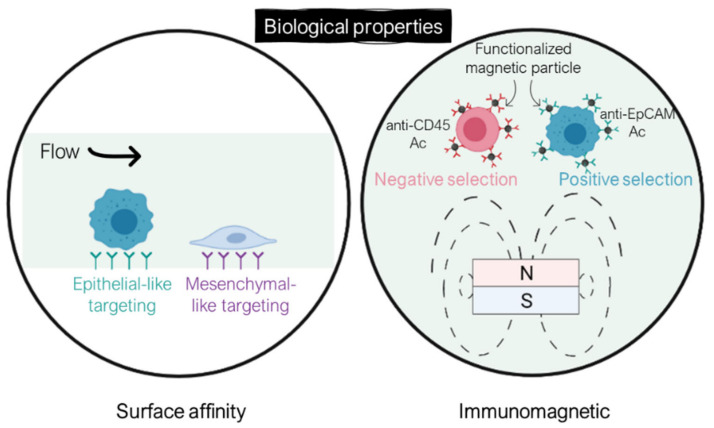
CTC-enrichment technologies based on their biological properties via antigen–antibody recognition through either surface functionalization or immunomagnetic separation using magnetic particles. Created with BioRender.com (Accessed on 31 January 2022).

The isolation of CTCs from other blood cells can also be achieved by exploiting biological properties of CTCs, such as their surface marker expression. These methods rely on the high specificity of the bonding between antibodies and expressed antigens in targeted cells. CTC isolation can be performed either by positive selection, where CTCs are collected as the target cell population, or negative selection, with WBCs as targeted cells. Biological-based separation methods can be categorized into either surface affinity approach through microchannel functionalization or the immunomagnetic approach using functionalized magnetic particles (Figure 6).

### 4.1. Surface Affinity Separation Methods

**Figure 7:**
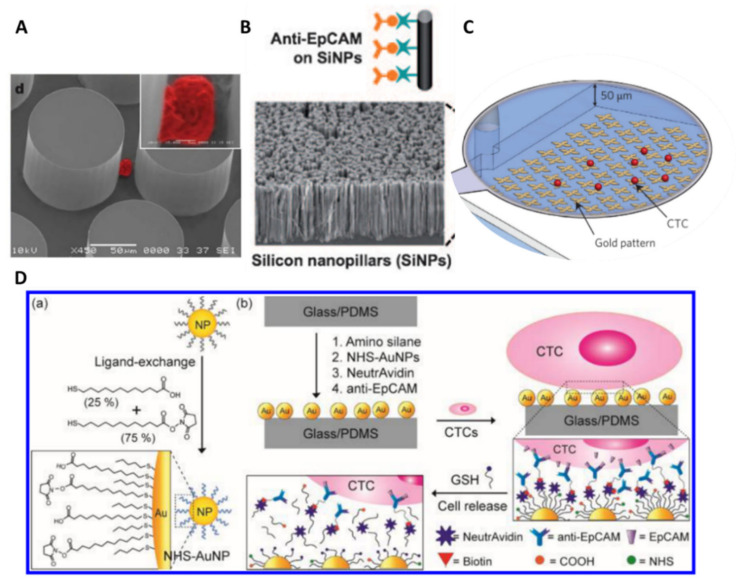
Surface affinity-based separation technologies. (**A**) CTC-Chip. CTCs are trapped on micropillars functionalized with anti-EpCAM antibodies. Reprinted by permission from [93]. (**B**) NanoVelcro chip. Silicon nanopillars are coated with anti-EpCAM antibodies. This strategy takes advantage from the nano-roughened surface of the NP assemblies to increase contact between CTCs and immobilized antibodies. Reprinted with permission from [97]. (**C**) GO Chip. Graphene oxide nanosheets are adsorbed onto the gold pattern and functionalized with anti-EpCAM antibodies. Reprinted (adapted) by permission from [98]. (**D**) Tuned ^HB^CTC-Chip for CTC release. (a) Thiol-functionalized gold nanoparticles (AuNPs). (b) Chip surface coating with AuNPs for CTC capture. In the presence of excess thiol molecules (GSH), the original thiol ligands with immobilized antibodies on the surface of the AuNPs can be exchanged with GSH molecules. Based on this thiol exchange reactions, captured CTCs can be detached from the chip surface, as represented in (b). Reprinted with permission from [99]. Copyright 2017 American Chemical Society.

The very first geometrically patterned microfluidic device with antibody-coated surfaces is the CTC-Chip, reported by Toner’s group in 2007 [93]. The device consists of an array of 78 000 anti-EpCAM-coated micropillars (100 µm in diameter, spaced by 50 µm) (Figure 7A). The array was arranged such that every three rows form an equilateral triangular to favor collisions between CTCs and functionalized micropillar surfaces. They obtained recovery efficiencies comprised between 74% and 80% for various cancer lines at a flow rate of 1 mL/h. The CTC-Chip was successfully tested for clinical samples with 99% sensitivity (115 out of 116) in the blood of patients with metastatic lung, prostate, pancreatic, breast, and colon cancer, with a purity of 50%. In addition, the chip enabled CTC isolation in 7/7 patients with early-stage prostate cancer. Toner’s group later reported an enhanced CTC isolation platform, the herringbone-chip (or ^HB^CTC-Chip), integrating herringbone grooves on the roof of the anti-EpCAM-coated microchannel [94]. These structures generate microvortices which enhance CTC capture through chaotic mixing and increased contact time between flowing cells and the antibody-functionalized surface. In comparison with the CTC-Chip, the ^HB^CTC-Chip allowed for higher sample throughput and increased CTC capture efficiency and purity. A capture efficiency of 92% on spiked cancer cells was achieved at 1 mL/h, with a 5% better purity. Clinical use of ^HB^CTC-Chip was further established and enabled the determination of CTC signaling pathways by RNA sequencing [95], identification of dynamic changes in CTC phenotypes [96], and investigation of the metastatic role of CTC clusters [49]. 

The use of nanostructured substrates, such as silicon nanopillars (NanoVelcro Chip) [97] (Figure 7B) or graphene nanosheets (GO Chip) [98] (Figure 7C), was also reported in microsystems to enhance CTC isolation sensitivity as nanomaterials offer high surface area-to-volume ratio and similar size to cellular surface components (e.g., microvilli and filopodia) [100]. However, the irreversible capture of CTCs on these nanostructures significantly limits downstream analyses and subsequent cell culture. Various approaches have been investigated to release CTCs after their isolation, using either thermosensitive polymers [101,102,103] or enzymatic degradation [104]. Nevertheless, thermoresponsive substrates require additional equipment to precisely control the temperature, while the use of enzymes, such as alginate lyase, may compromise the viability of CTCs due to over exposure to the degraded film itself and the enzymatic solution. Recently, Stott’s group engineered the surface of the ^HB^CTC-Chip with a gold nanoparticle coating and utilized a thiolated ligand-exchange reaction to isolate and release CTCs from whole blood [99]. Indeed, metal–thiol interactions can be disrupted in the presence of excess thiol molecules (i.e., glutathione), leading to an exchange between the original ligands with immobilized antibodies and the thiol molecules, resulting in the release of cancer cells from the surface (Figure 7D). This strategy takes also advantage of the nano-roughened surface of the NP assemblies to increase contact between CTCs and immobilized antibodies, therefore enhancing capture efficiency. This new NP-functionalized chip achieved a capture efficiency as high as 99% for epithelial cancer lines, with lower nonspecific binding compared with their previous ^HB^CTC-Chip (35% decrease). For non-epithelial cancer lines, a cocktail of antibodies had to be used within the chip to increase capture efficiency, from 16% to >90%. In addition, the chip successfully released 90% of the captured cells that were further cultured for 5 days with a preserved viability (78–87%). 

Similarly, Tseng’s group reported the tuning of their NanoVelcro Chip with a phenylboronic acid (PBA)-grafted PEDOT nanosubstrate to release captured CTCs upon exposure to a glycan molecule (i.e., sorbitol) [105], which has a stronger affinity to PBA. CTCs were isolated from the blood with patients with prostate cancer (PCa) and purified by this PEDOT NanoVelcro chip. The chip provided well-preserved RNA transcripts for the analysis of the expression level of several PCa-specific RNA biomarkers which may provide clinical insights into the disease.

The main advantage of this method, based on antibody–antigen reaction within functionalized microfluidic systems, is the high sensitivity for a given cellular type with a preserved viability. Nonetheless, lower throughput is achieved compared with physical-based separation approaches. In addition, special attention should be paid to enable the collection of captured CTCs. The main drawback of this method remains that, most of the time, a unique antigen is targeted (usually EpCAM), therefore limiting the recovery of heterogeneous CTCs.

### 4.2. Immunomagnetic-Based Separation Methods

The immunomagnetic separation relies on the conjugation of magnetic particles to cells via antigen–antibody recognition in order to confer them magnetic properties. When subjected to a non-uniform magnetic field, magnetically labelled cells can be manipulated within the microchannel for sorting applications [106,107,108]. This phenomenon is called magnetophoresis. Magnetophoresis offers a contactless manipulation, making this technique nondestructive for biological samples; it offers robustness, since this method is not sensitive to pH, temperature, etc.; finally, it offers tunability—the magnetic force depends on the particle size, the magnetic properties of the target, and the surrounding medium, as well as the gradient of the magnetic field.

The immunomagnetic separation can either target CTCs (positive selection) or WBCs (negative selection). The benefit of the negative selection over the positive one is the ability to collect all CTCs regardless their surface marker expression. Nonetheless, given the high concentration of WBCs in blood, their depletion is more challenging. 

Various strategies were implemented for the immunomagnetic separation of CTCs in a microfluidic device and will be further detailed. 

#### 4.2.1. Positive Selection 

**Figure 8:**
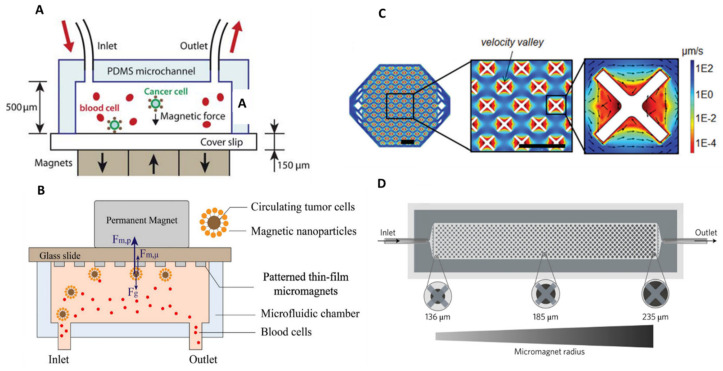
Magnetic sources for CTC isolation from macroscale to microscale. (**A**,**C**) From the use of an external permanent magnet to the integration of nickel microstructures within the microfluidic channel. These microstructures, acting like microtraps, achieved an average 18.4% increase in capture rate of magnetically labeled CTCs in comparison with the previous design. Reprinted with permission from [109]. Reproduced from [110] (http://creativecommons.org/licenses/by/4.0/) (Accessed on 27 October 2021). (**B**,**D**) Toward the combination of X-shaped velocity valleys as low-flow velocity regions with circular nickel microstructures as capture spots. This configuration achieved a >90% capture efficiency for cancer cell lines with various EpCAM expression levels and enabled them to be magnetically ranked, thanks to the gradual increase in nickel microstructure size. The capture of low-expression cells requires the action of larger nickel structures; therefore, it occurs in the later zones of the chip. Reproduced from [111] with permission from the Royal Society of Chemistry. Reprinted from [112] by permission from Nature.

Magnetophoresis, which, as introduced earlier, is the motion of an object in a non-uniform magnetic field, and coupled with microfluidic technology, can be used for sorting applications. Researchers have carried out hard work on the optimization of magnetic field gradient sources to reach high magnetic forces, therefore maximizing sorting efficiencies. For example, Hoshino’s group and Kelley’s group demonstrated the advantage of downscaling the size of the magnetic source. Historically, Hoshino et al. implemented a CTC sorting device using an array of three NdFeB block magnets (19 × 13 × 5.6 mm^3^) located at the bottom of the microfluidic channel. CTCs in blood were labelled with EpCAM-functionalized Fe_3_O_4_ magnetic nanoparticles and captured by the magnetic field as the blood flows through the microchannel [109] (Figure 8A). 

Later, Hoshino’s group highlighted the benefit of working with micrometer-sized magnets. Nickel (Ni) microstructures were integrated into the microfluidic channel which act like microtraps [110] (Figure 8B). Arrayed Ni microstructures were first defined by standard photolithography and next, a thin film of a nickel layer (250 nm thick) was deposited by thermal deposition (on top of a 15 nm-thick chromium adhesion layer). These nickel microstructures with the dimensions of 20 µm × 20 µm were designed to be compatible with CTC diameter. In total, about 8750 magnetic traps were integrated on the chip, i.e., 25 traps/mm^2^ [113]. They were magnetized upon application of a magnetic field which is supplied by the same configuration as their previous chip, i.e., three NdFeB block magnets. With this new design, they achieved an average 18.4% increase in capture rate in comparison with their previous configuration, where the magnetic field was generated by external magnets only. In addition, they observed improved working stability with the nickel microconcentrators as the capture rate variability was lowered. The average capture rate with nickel-patterned microstructures was 97.3% at a flow rate of 2.5 mL/h. Subsequent immunofluorescence staining and FISH analyses were performed by fixing captured cancer cells on the channel substrate. Furthermore, they studied the trapping distribution within the chip according to the position of the permanent magnets and nickel microtraps. The median capture position was located on the front edge of the permanent magnets array, indicating that the permanent magnets provide the major attractive forces. Besides, the total ranges of cell distribution area increased, which demonstrates the additional magnetic trapping sites of the nickel microstructures, therefore preventing cell aggregation issues. Finally, they clinically verified the trapping ability of the device by screening blood samples from patients with metastatic cancers (colorectal, lung, prostate, and breast cancers) and found between 1 and 215 CTCs in screened patient samples (blood volume ranging from 5 to 10 mL).

Similarly, Kelley’s group developed a more complex device, integrating X-shaped microstructures as capture spots [111]. These capture structures generate regions of locally low flow velocity (Figure 8C)—termed velocity valleys—so that the magnetic force, resulting from an external, millimeter-sized magnet, is sufficient to overcome the lowered drag force. Cancer cells, which are labelled with anti-EpCAM magnetic nanoparticles, entering the valley, will get captured. Furthermore, they devised successive zones with increasing channel cross-section to decrease the average linear velocity and thus the drag force. Doing so, they managed to capture cancer cells as a function of EpCAM expression by studying their trapping location. They later upgraded their “velocity valley” design by integrating round nickel microstructures centered on their X-shaped capture spots [114]. These microstructures were first patterned using standard lithography processes, and then covered with a 1.5 µm-thick Ni layer by sputtering. These Ni microstructures increase in radius along the length of the channel, from 136 to 235 µm, generating 100 discrete zones. Each of the 100 zones has two rows of X-structures with the same Ni structure diameter. This gradual increase in the magnetic capture sites exposes the magnetically labeled cancer cells to enhanced magnetic field gradients at the edges of the Ni traps, enabling them to be magnetically ranked, regarding their surface marker expression (magnetic ranking cytometry device, MagRC) (Figure 8D). The capture of low-expression cells requires the action of larger nickel structures; therefore, it occurs in the later zones of the chip. This combination of low flow and high magnetic field gradients leads to a >90% capture efficiency for cell lines with various EpCAM expression, at a flow rate of 0.5 mL/h. In comparison, their previous velocity valley chip reached similar performances, but by tuning the flow rate for each cell type. At a flow rate of 0.5 mL/h, 40% of SKBR3 cells were captured. They later achieved successful profiling of CTC phenotypes in clinical samples [112]. They observed that patients with localized prostate cancers presented a greater phenotype diversity than patients with metastatic prostate cancer. Recently, Kelley’s group implemented their MagRC device for the tracking of the expression of therapeutic protein targets in CTCs [115]. This was achieved using magnetic cell-labelling reagents that can target intracellular proteins, and, therefore, enabling magnetic ranking of CTCs according to the expression levels of intracellular proteins. By measuring these protein levels within isolated CTCs and analyzing these protein markers at the single-cell level, they could identify drug targets or predict therapeutic response.

**Figure 9:**
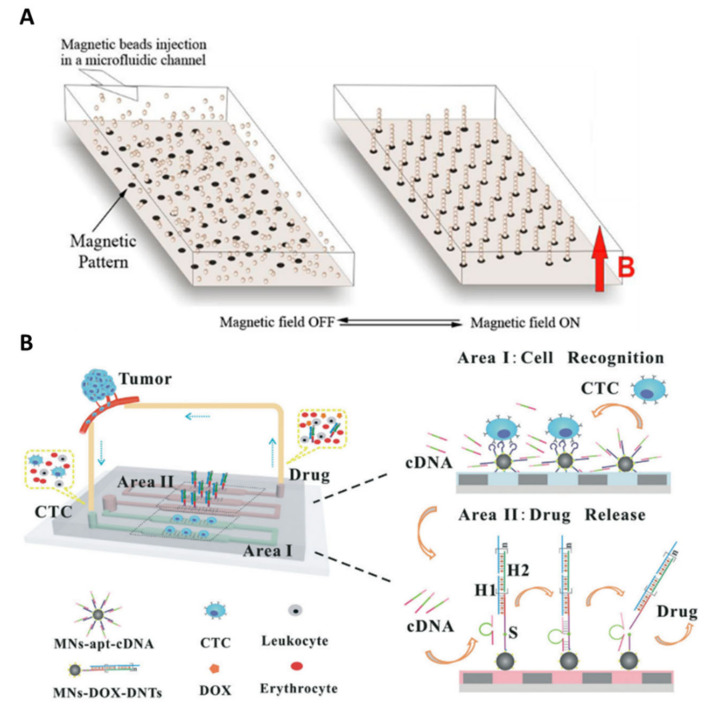
Ephesia technology for CTC isolation. (**A**) Self-assembly of anti-EpCAM functionalized magnetic beads along the microchannel height, which act as traps for CTCs. Columnar bead arrays were localized by microcontact printing of a magnetic pattern made of ferrofluid. Captured CTCs can be released by removing the external permanent magnet. Red letter B represents the magnetic field as a “magnetic field ON”. Reprinted with permission from [116]. Copyright 2010 American Chemical Society. (**B**) Arrangement of functionalized magnetic nanospheres within the microchannel through the use of a nickel patterns. The liquid-biopsy-guided drug release system (LBDR system) consists of two areas loaded with two types of functionalized magnetic nanospheres (MNs). Tumor cells are first recognized and captured by EpCAM-aptamer-functionalized MNs (Area I) which leads to the release of corresponding complementary strands (cDNAs), due to the conformational change of the aptamers. cDNAs present cleaving capability, which could trigger a subsequent doxorubicin (DOX) drug release process (Area II). Reproduced from [117] with permission from the Royal Society of Chemistry.

Furthermore, other various immunomagnetic-based approaches have been implemented for CTC-positive selection. Viovy’s group reported the Ephesia technology, which consists of self-assembled anti-EpCAM functionalized magnetic beads forming columnar arrays along the microchannel height and acting as a trap for target cells (Figure 9A). They first proposed to use a permanent magnetic pattern with the desired organization, deposited at the bottom of the microchannel, to direct bead self-assembly [116]. This method is based on the microcontact printing of a water-based ferrofluid onto glass, to localize and organize the functionalized beads columns in the channel. They demonstrated a capture efficiency as high as 94%, and the possibility to cultivate, in situ, the captured cells. Clinical samples issued from patients with B-cell hematological malignant tumors (leukemia and lymphoma) were also characterized after CTC isolation. Phenotype and morphology analyses, as well as intranuclear high resolution imaging, were conducted within the chip.

Magnetic columns must be tightly anchored to the bottom layer of the chip to stand firm against hydrodynamic flow during the whole capture and analysis. They later proposed a capillary assembly technique [118], using a microstructured PDMS template with micron-sized well patterns, to improve the stability of the bead columns [119]. Similarly, Zhang’s group reported the use of micrometric nickel squares as a magnetic pattern to control the arrangement of anti-EpCAM-coated magnetic nanospheres (MNs) within the microchannel [120]. The 9 µm-thick nickel microstructures were obtained by electroplating and encapsulated in a 2 µm-thick PDMS film [121]. When nickel patterns are magnetized through the presence of external permanent magnets, they generate a high magnetic field gradient around them, resulting in the capture of magnetic beads at their edges. Interestingly, captured CTCs could be recovered after removal of the permanent magnets. This magnetically controlled microfluidic device was further implemented for a liquid-biopsy-guided drug release system to capture CTCs and accordingly release an appropriate amount of anticancer drug [117]. This system consisted of two areas loaded with two functionalized MNs: recognition MNs for CTC capture, and drug-loaded MNs for drug release (Figure 9B). Cancer cells are recognized and captured by EpCAM aptamers on recognition MNs which then triggers the release of complementary strands inducing a subsequent drug release. Thus, drugs were released according to the number of captured CTCs, and different levels of treatment could be implemented according to the malignant progression of cancers. The novelty of this device is the combination of cancer diagnosis and therapeutic functions and may help in the development of personalized cancer medicine. Another application was investigated by Zhang’s group which relies on intravenous collection followed by in vivo CTC detection and monitoring. Blood was extracted from a mouse’s blood vessel and then introduced into the separation device [122]. Similarly, Nagrath’s group implemented their immunoaffinity-based ^HB^GO chip for ex vivo capture of intravenously infused CTCs in canine [123].

##### Phenotypic Heterogeneity Tracking

**Figure 10:**
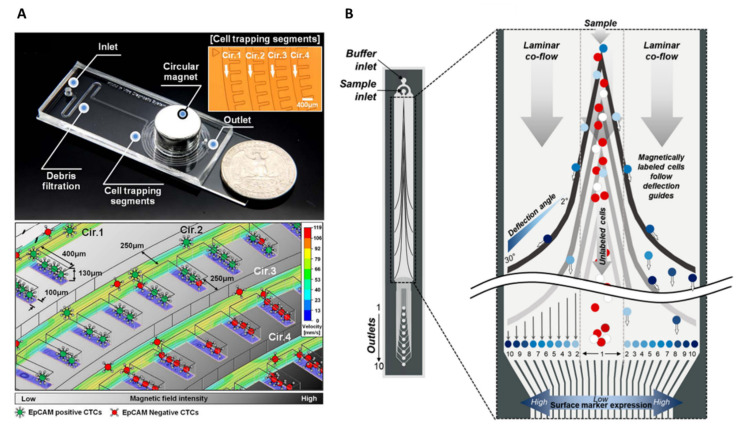
Heterogeneity tracking in immunomagnetic-based separation systems. (**A**) Spiral shape design can gradually decrease the distance to the center circular shape permanent magnet. Heterogeneous CTCs specifically position in trapping segments regarding the number of anti-EpCAM-conjugated magnetic nanoparticles on their surface. Low-expression cells will be captured in the center of the spiral channel where the distance to the external permanent magnet is small. Reprinted from [124], with permission from Elsevier. (**B**) Prismatic deflection separates a continuous CTC sample stream into discrete subpopulations based on CTC surface marker expression level. Co-based ferromagnetic guides are made up of distinct segments having angles ranging from 2 to 30° and, in the presence of an external magnetic field, induce a lateral deflection of a magnetically labeled target. The angle of the deflection guides relative to the direction of flow dictates the direction of the magnetic force, while the amount of magnetic loading on the surface of the cell dictates its magnitude. Reprinted with permission from [125]. Copyright 2018 American Chemical Society.

Nevertheless, the approaches cited above do not take into account the surface marker expression heterogeneity in CTCs. Special designs were imagined to track this heterogeneity in immunomagnetic separation-based systems for CTC sorting. Kwak et al. reported the fabrication of a spiral-shaped channel capable of capturing magnetically labeled CTCs by magnetophoresis regarding their EpCAM expression level [124]. This was achieved thanks to the spiral shape design that can gradually decrease the distance to the center circular shape permanent magnet (external to the microsystem), resulting in specific positioning of heterogeneous CTCs depending on the number of anti-EpCAM-conjugated magnetic nanoparticles on their surface (Figure 10A). CTCs with high EpCAM expression will be captured in cell trapping segments located along the first channel loop while CTCs with low EpCAM expression will travel along successive channel loops to finally get trapped where the distance between the circular channel and magnet is small. Aldridge et al. reported the Prism Chip, a more complex design using variably angled ferromagnetic guides (magnetized by an external neodymium magnet) to induce prismatic deflection of magnetically labeled CTCs and separate them into distinct subpopulations, corresponding to their EpCAM expression levels [125] (Figure 10B). Analogously to the functioning of an optical prism dispersing light into its component wavelengths, this approach separates a flowing stream of cells into discrete fractions. They achieved a recovery efficiency of 88% at a flow rate of 30 mL/h, and improved purity by performing a first prismatic deflection of WBCs using magnetic nanoparticles conjugated to anti-CD45 and anti-CD15 antibodies. They integrated a graphene Hall sensor array to enumerate the isolated cell subpopulations, including cell clusters. The Hall sensor array consists of patterned graphene crosses on which titanium (10 nm) and gold (50 nm) contacts were deposited using electron beam evaporation. Magnetically labeled cells flowing over the sensor array induce a change in magnetism, proportional to the cell’s magnetic loading, which is converted into a voltage difference. Heterogeneous cells, such as single CTCs or CTC clusters (with more surface biomarkers due to the larger surface area), can therefore be differentiated without requiring the whole equipment needed for fluorescence microscopy, offering low-footprint solutions for cancer diagnosis.

#### 4.2.2. Negative Selection

**Figure 11:**
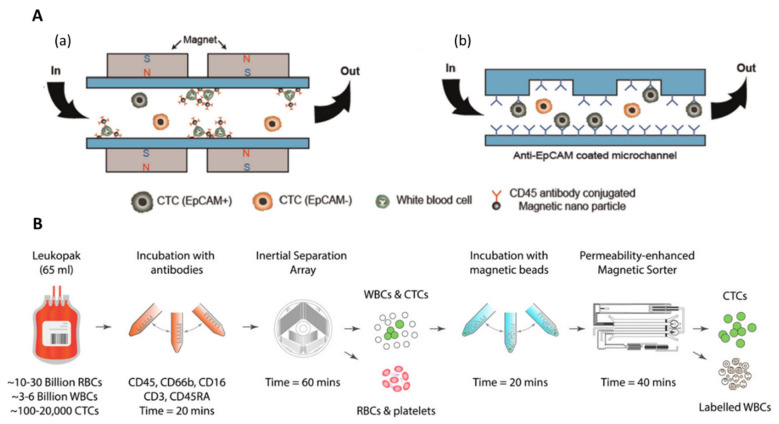
Tumor-marker-independent selection. (**A**) Two-stage microfluidic chip for negative selection of CTCs. (**a**) Magnetically labeled WBCs are first eliminated in the first immunomagnetic stage and (**b**) CTCs are then selectively isolated based on their surface marker expression in the anti-EpCAM-coated chip region. Reprinted from [119], with permission from Elsevier. (**B**) Whole workflow for high-throughput CTC separation from full (65 mL) leukapheresis samples. RBCs and platelets are first removed from leukapheresis products using size-based inertial separation, followed by immunomagnetic removal of WBCs, which were labeled prior with a mixture of biotinylated antibodies targeting the pan-leukocyte cell surface antigens. CTCs were recovered without relying on antigen markers. Reprinted with permission from [128] under the Creative Commons Attribution License 3.0.

Other immunomagnetic-based strategies relying on WBC depletion (negative selection) were also studied. Such tumor–antigen independent immunomagnetic separation methods were investigated to overcome marker expression variability among CTCs. By specifically removing WBCs, typically using anti-CD45 antibodies, CTCs can further be collected for downstream analysis. These approaches offer an opportunity to isolate CTCs regardless of their phenotype and ensure that CTC viability is maintained. Hyun et al. fabricated a two-stage microfluidic chip (μ-MixMACS chip) for negative selection of CTCs [126,127]. The microfluidic chamber, with a height of 930 µm and a total volume of 1 mL, was sandwiched between 2 magnet array cartridges. The magnet array, which consisted of millimeter-sized rectangular NdFeB magnets, arrayed in a laser-cut plastic cartridge, generated magnetic fields parallel to the flow direction for WBC depletion, with strong magnetic field gradients located between two adjacent magnets. In the first stage, WBCs labeled with CD45 antibody-conjugated magnetic nanoparticles were depleted inside the chip by magnetophoresis while CTCs exited through the outlet. Cells are then focused in the center of the channel by inertial forces and entered the second stage in which CTCs were specifically captured on the antibody-coated (e.g., EpCAM or HER2) channels (Figure 11A). They isolated tumor cells based on their surface marker expression levels on the anti-EpCAM antibody-coated chip and anti-HER2 antibody-coated chip and achieved capture efficiencies of 98.91% for EpCAM+ cells and 86.51% for HER2+ cells, respectively, with 22% purity, at high throughput (24 mL/h). Nonetheless, a limitation of this approach is the risk of channel clogging for high-capacity isolation.

Large volumes of blood have to be processed to ensure the collection of a sufficient number of CTCs, which might cause clogging due to the large number of WBCs per 1 mL of blood (about 10^6^). Recently, Mishra et al., reported an ultra-high-throughput magnetic sorting chip, the ^LP^CTC-iChip, which processed very large blood volumes (65 mL) for negative selection of CTCs [128]. By combining soft-iron-filled channel—to intensity the field gradient within sorting channels—with inertially focused streams of cells, they achieved massive depletion of magnetically labeled WBCs. CTCs and WBCs were collected in two separated outputs. They obtained an 86% recovery efficiency with 99.97% of depleted, resulting in an average purity of 0.3% at a remarkable flow rate of 168 mL/h. This magnetic device was used after a previous non-equilibrium inertial separation array [129], which removes RBCs and platelets based on their small physical size, compared with nucleated cells (Figure 11B). From this prior, physical-based isolation step, they depleted >99.999% RBCs and >99.999% platelets. 

#### 4.2.3. Hybrid Separation Devices: Combination with Physical-Based Separation Technologies

It can be of first interest to combine immunomagnetic separation with other physical-based approaches. The combination of the two approaches can compensate the inherent drawback of each technique, enabling the detection of a wider range of tumor cells exhibiting different properties among them. Most multi-step isolation methods can be divided into pre-enrichment and isolation steps. The pre-enrichment part is usually based on a label-free method that allows for continuous CTC enrichment [130,131].

**Figure 12:**
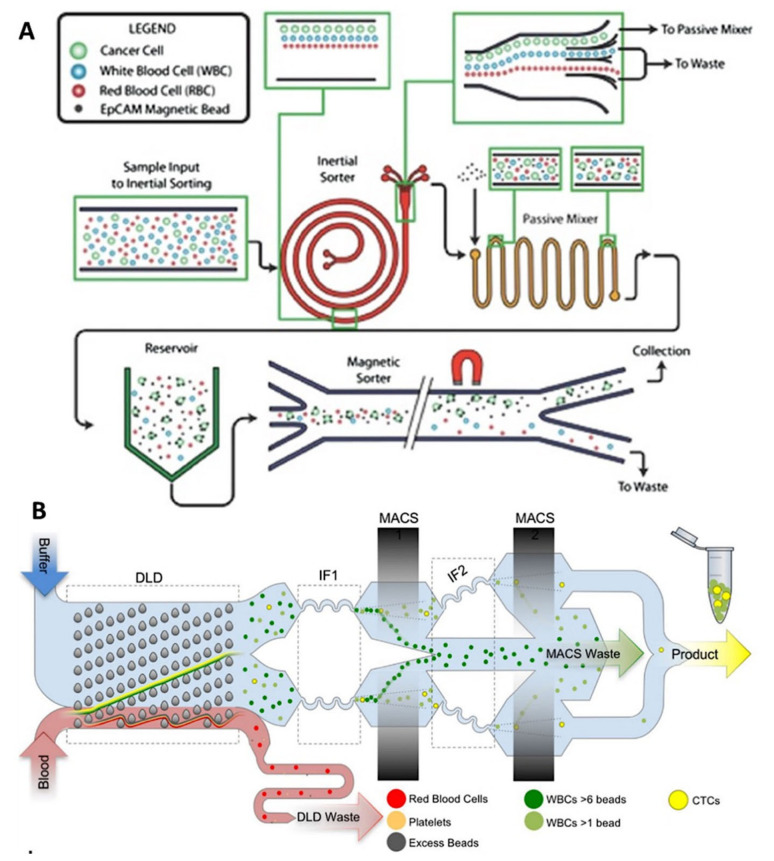
Integrated separation devices combining a size-based pre-enrichment step and an immunomagnetic-based purification step. (**A**) Integration of inertial sorter and magnetic sorter modules. Complete RBCs removal and partial WBC depletion through an inertial separation step in a spiral-shaped microchannel, followed by immunomagnetic separation of magnetically labeled CTC. The labeling step of CTCs with anti-EpCAM-coated magnetic beads is performed on-chip. The magnetic sorting step enabled the distinct isolation of CTCs according to their EpCAM expression levels by adjusting the distance of the external magnet from magnetic particles flowing in the sorter. Reprinted with permission from [133] (https://creativecommons.org/licenses/by/4.0/) (Accessed on 27 October 2021). (**B**) CTC-iChip technology. RBCs and platelets are first removed by deterministic lateral displacement and remaining CTCs and magnetically labeled WBCs then enter two successive inertial focusing/magnetic sorting stages for WBC depletion. Reprinted with permission from [135] (https://creativecommons.org/licenses/by/4.0/) (Accessed on 27 October 2021).

Nagrath’s group reported a two-step isolation method: the first pre-enrichment stage consists of Dean flow fractionation in a spiral channel to remove RBCs and most of WBCs, and the second isolation step is performed by magnetophoresis on magnetically labeled CTCs [132]. Contrary to most separation devices, CTC labeling is conducted on-chip, in a passive mixer, where EpCAM-coated magnetic beads and CTCs are infused at 100 µL/min, following a 5 min on-chip incubation in reservoirs to promote antibody–antigen interactions (Figure 12A). The magnetic sorter module enables the distinct isolation of CTCs according to their EpCAM expression levels by adjusting—on the micron scale—the distance of the external magnet from magnetic particles flowing in the sorter. The magnetic field strength experienced by the labeled cancer cells could thereby be tuned, and as cell magnetic loading depends on their surface marker expression, cells could be specifically separated. The device achieved a 90% recovery efficiency on a spiked cell line, with between 82 and 801 contaminating WBCs/mL, resulting in purities of up to 75%. The clinical utility of the device was demonstrated by processing pancreatic ductal adenocarcinoma blood samples from 6 patients with and characterizing the isolated CTCs from these samples. Tumor cells were isolated based on low, moderate, and high EpCAM levels. This platform enables the comparison of tumor cell subpopulations and further investigation should help identifying the impact of cell heterogeneity on patient outcomes and tailoring therapeutic targets for virulent cell subpopulations. Besides, the ultra-pure isolation of CTCs from patient blood samples enabled highly specific molecular profiling of patient CTCs [133]. More recently, Nagrath’s group developed an original inertial device, the CTCKey™ [134], to isolate CTCs at high throughput, without any dilution or use of sheath buffers—usually required for inertial based separation methods. The concentrated product thus obtained can be further processed using any CTC isolation device.

Toner’s group first reported the CTC-iChip which combinates three different antigen-independent principles for CTC isolation: deterministic lateral displacement, inertial focusing, and magnetophoresis [135] (Figure 12B). The individual components previously manufactured using deep reactive ion silicon etching and PDMS soft lithography [136] were integrated on a single mass-produced plastic chip, improving the accessibility of the CTC-iChip technology. Whole blood is injected within the monolithic chip and passes through a first DLD separation step, after which, RBCs and platelets are removed. The remaining CTCs and magnetically labeled WBCs then enter two successive inertial focusing and magnetic sorting stages for WBC depletion. Magnetic field gradients were generated by four magnets, arranged in a quadrupole configuration and housed in a custom aluminum manifold. The sensitivity of the first stage enables the removal of labeled WBCs with more than 6 magnetic beads on their surface. The remaining cells enter the second stage, which removes cells that are labeled with at least 1 magnetic bead. The performances of the chip were characterized across 11 different cell lines, and the chip achieved a remarkable median recovery of 99.5%, with a high purity (445 WBCs/mL). In particular, they highlighted the importance of performing negative depletion of blood cells as they found that neither CTC size nor EpCAM expression can maximize isolation efficiency as many CTCs found were small and expressed lower levels of EpCAM. In addition, they found that both parameters were significantly dependent on the individual patient and widely variable within a single patient. These results will help guide the design of future CTC isolation and diagnostic strategies based on negative depletion of blood cells. 

### 4.3. Summary of Biological-Based Separation Methods

The advantages and limitations of both biological-based separation methods can be found in Table 4. Despite the required labeling step, the immunomagnetic separation can achieve high sensitivity and specificity, due to the antigen–antibody reaction and magnetic contrast supplied by conjugated magnetic particles, while providing higher throughput and a certain compatibility with downstream analysis. In addition, this approach has proven to be versatile given the various magnetophoretic strategies implemented. 

Finally, the performances of the reported biological-based separation technologies are summarized in Table 5 and Table 6, for cell line studies and clinical studies, respectively.

## 5. Downstream Characterizations to Unveil CTC Clinical Significance

Microfluidic-based isolation devices should not only provide relevant sorting efficiencies but should also be compatible with downstream characterizations, since further CTC study has shown significant clinical potential. On one hand, the detection of CTCs can provide clinical information on the tumor stage and can be used in early cancer diagnosis and disease prognosis [137,138,139,140]. On the other hand, CTCs are good surrogate biomarkers for treatment efficacy monitoring, enabling a personalized therapeutic approach [141,142]. Indeed, it became clear over time that the “one drug fits all” treatment model was limited; hence, is being replaced by personalized medicine. As reported above in Table 3 and Table 6, various downstream characterizations were implemented after microfluidic-based CTC isolation: from (i) immunofluorescence-based CTC enumeration to (ii) phenotypic profiling, (iii) molecular characterizations, and (iv) CTC culture. A common and widespread subsequent study consists of determining CTC counts within patient samples by an immunofluorescence assay. Indeed, the prognostic significance of CTCs has been previously demonstrated in numerous studies on patients at early disease stages without clinical and radiological signs of overt metastases, particularly in breast cancer [140,143,144], but also in other tumor entities [145,146]. After CTC enrichment using microfluidic-based isolation technologies, CTCs are immunofluorescently stained with EpCAM/cytokeratin/vimentin inclusion markers and CD45 exclusion marker (WBC staining) [15,16,17,115,128]. Beyond prognostic and diagnostic applications, further phenotypic profiling can be performed to specifically identify therapeutic targets [81,147]. The identification of drug-specific protein biomarkers can also be determined by cancer genome sequencing which consists in screening recurrent genes in different cancer types, named “oncodriver” genes, or oncogenes. Often mutated and overexpressed in cancers, oncogenes play an essential role in cancer progression since they promote cell proliferation and resistance to apoptosis. CTCs can therefore be screened for genetic mutations in known oncogenes. Microfluidic-based isolation technologies should therefore be compatible with molecular analysis workflow to widen clinical applications. Workflow compatibility for qPCR [71,105,133], Sanger sequencing [17], or next-generation sequencing [99] were reported. In addition, FISH techniques, such as ALK and HER-2 amplification, can be accomplished on harvested CTCs to probe DNA aberrations and implement adapted treatment strategies [15,16,110,148]. Last but not least, therapy efficacy monitoring can be performed by in vitro culture of enriched CTCs to explore drug susceptibility testing [149,150,151,152].

## 6. Conclusions

In the context of liquid biopsy and personalized cancer medicine, researchers have shed light on CTCs as biomarkers for cancer diagnosis, prognosis, and monitoring. Nevertheless, there is still a lot of ongoing work to tackle the challenges raised by their isolation, given their rarity among other blood cells, their phenotypic and size heterogeneities, and the need to preserve their viability for downstream analysis. Over the past decade, microfluidic devices have been shown to have promising features for addressing these challenges, and studies are still being widely conducted to keep providing the best performances for CTC isolation, including high throughput, purity, recovery, and clinical relevance. In particular, the reported microfluidic-based separation technologies—based on either the physical or biological properties of CTCs—have highlighted the importance of achieving both the high recovery of CTCs and the high purity, which can be arduous due to the above-mentioned CTC-inherent challenges. Nevertheless, microfluidic devices have made a great breakthrough towards their implementation in clinical studies, and several commercialized technologies are now awaiting FDA clearance (ClearCell FX1, VTX-1, Parsortix, etc.).

Integrated microfluidic systems, as reported with combined magnetophoresis with other size-based separation methods, for example, have emerged as next-generation CTC isolation systems, since they can offer the following: (i) effective recovery of CTCs and CTCs clusters simultaneously; (ii) ultra-pure samples with minimal contamination of normal blood cells; (iii) high-throughput sorting with preserved viability. The widespread use and development of microfluidic systems, aimed at supporting liquid-biopsy-based applications, will represent a paradigm shift for cancer clinical care. In particular, the detection, enumeration, and characterization of CTCs will play a significant role in clinical applications involving early detection of aggressive cancers, selection of therapies, identification of drug resistance, and discovery of novel therapeutic targets. Tremendous research efforts have already been made within the past decade to provide robust CTC separation methods and the next generation of devices will certainly provide a complete workflow integrating both isolation and characterization (culture, drug testing, etc.) steps into a single chip.

## Figures and Tables

**Table 1:** Advantages and limitations of physical-based separation methods for CTC isolation.

Separation method	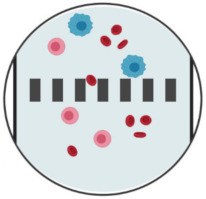 Microfiltration	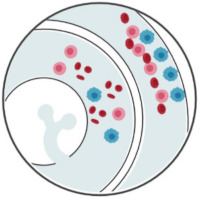 Hydrodynamics	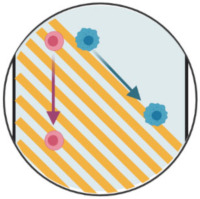 Dielectrophoresis
Separation criteria	Size, deformability	Size	Size and dielectric properties
Pros	Easy approach, high throughput, label free	High throughput, label free, CTC recovery	Label free, CTC recovery
Cons	Risk of clogging, low purity, challenging downstream analysis	Low purity	Low throughput, separation is limited over time, specific cell type, electric field frequency and buffers are required

**Table 2:** Performances of reported physical-based separation technologies in cell line studies.

Separation Method	Technology	Selection Criteria	Throughput	Sample Composition	Recovery	Viability	Purity	WBC Depletion	Enrichment Factor	Refs.
Microfiltration	Microellipse filters	Size (5–18 µm)	1 mL/h	MCF-7, HepG2, and HeLa cells in 1 mL PBS supplemented w/1% BSA and 0.05% tween-20 ^†^	>90%	90%	--	--	--	[30]
Microfiltration	Microfluidic ratchets	Size (6 µm), deformability	1 mL/h	UM-UC13 cells in 5 mL whole blood	77–90% (various cell lines)	99%	--	--	8500	[29]
Microfiltration	Rectangular MCA	Size (8 µm)	12 mL/h	NCI-H358, NCI-H69, and NCI-H82 cells in 1mL whole blood	80–90%	98%	76–78%	--	7000	[45]
Microfiltration	Pyramidal MCA	Size (8 µm)	6 mL/h	MCF-7, SW620, and HeLa cells in 1 mL whole blood	76–84%	--	--	99.9985%	--	[46]
Microfiltration	Parsortix™	Size (10 µm)	10 mL/h	PANC-1, PC3, A375, A549, and T24 cells in 2 mL whole blood ^†^	42–70% *	99%	~60%	~99.96%	--	[41]
Hydrodynamics (DLD)	Triangular posts	Size (7 µm)	600 mL/h	MDAMB231, PC3, and MCF10A cells in 1 mL diluted blood	>85%	≥95%	--	--	--	[54]
Hydrodynamics (DLD)	Asymmetric pillars	Size (>30 µm, CTC clusters)	0.5 mL/h	Ex vivo cultured breast cancer clusters in whole blood	98.7 ± 2.4% (large clusters) 65.5 ± 6.5% (small clusters)	91.7 ± 2.5%	--	1.58 ± 0.13 log (stage 1) and 2.48 ± 0.22 log (stage 2)	--	[56]
Hydrodynamics (inertial focusing)	Multi-flow straight channel	Size (15 µm)	1.2 mL/h	HCC827 and H460 in 5 mL diluted blood ^†^	>93%	--	88.7%	--	--	[58]
Hydrodynamics (DFF)	ClearCell	Size (14 µm), deformability	36 mL/h	T24, MCF-7, and MDA-MB-231 cells in lysed and 2× concentrated blood in PBS ^†^	80.3 ± 7.9%	87.5%	--	~99.99% 4 log	--	[69,70]
Hydrodynamics (DFF)	Labyrinth (spiral channel w/sharp corners)	Size, deformability	150 mL/h	MCF-7, PANC-1, PC-3, and H1650 cells in buffer or whole blood	>90%	High	--	>4 log	--	[71]
Hydrodynamics (Microvortices)	Vortex HT	Size (13 µm), deformability	480 mL/h (10× diluted blood) or 48 mL/h (whole blood)	MCF-7 cells in 4 mL 10× diluted blood	84%	83.9 ± 4.0%	>80%	4–5 log	--	[60]
Dielectrophoresis	Apostream™ (DEP-FFF)	Size and dielectric properties	~1 mL/h	SKOV3 and MDA-MB-231 cells in 1 mL buffer ^†^	68.3 ± 10.4%	97.6%	~0.3%	99.33 ± 0.56%	--	[78]
Dielectrophoresis	ODEP	Size and dielectric properties	24 µL/h	PC-3 cells in sucrose solution	54 ± 7%	--	94.9 ± 0.3%	--	--	[86]

^†^ Cancer cells were spiked in low numbers (below 100 cells) to meet clinically relevant cell numbers. * With the Parsortix™, captured cancer cells could be harvested from the device for further downstream analyses. Harvest efficiency ranged from 27% to 40%.

**Table 3:** Performances of reported physical-based separation technologies in clinical studies.

Separation Method	Technology	Blood Sample Volume	Cancer Type	Number of CTCs	Detection Sensitivity	Remaining WBCs/mL	Downstream Analysis	Refs.
Microfiltration	Microellipse filters	2–3 mL	Metastatic (M)-breast cancer (*n* = 4), Colon (*n* = 1), NSCLC (*n* = 12)	1–10/2–3 mL, 6–10/2–3 mL, 1–20/2–3 mL	100% (17/17)	--	Immunofluorescence staining and enumeration	[30]
Microfiltration	Microfluidic ratchets	2 mL	M-castrate-resistant prostate cancer (*n* = 20)	Median 178/7.5 mL	95% (19/20)	--	Immunofluorescence staining and enumeration	[29]
Microfiltration	Rectangular MCA	2–4 mL	SCLC (*n* = 16)	1–73/mL (Median 2/mL)	100% (16/16)	854 ± 306 (Median)	Immunofluorescence staining and enumeration	[45]
Microfiltration	Pyramidal MCA	1–3 mL	Breast (*n* = 3), Lung (*n* = 3)	23–86/mL, 0–48/mL	83% (5/6)	396–3845	Immunofluorescence staining and enumeration	[46]
Microfiltration	Parsortix™	4 mL	Breast (*n* = 10), Colon (*n* = 10), Lung (*n* = 6)	0–3/mL, 0–1/mL, 0–7/mL	38% (10/26)	--	Immunofluorescence staining and enumeration, molecular characterization (RT-PCR and array-based comparative genomic hybridization)	[41]
Hydrodynamics (Inertial focusing)	Multi-flow straight channel	2 mL	M-NSCLC (*n* = 8)	Median 12/mL	75% (6/8)	--	Immunofluorescence staining and enumeration	[58]
Hydrodynamics (DFF)	ClearCell	7.5 mL	Lung (*n* = 15), Breast (*n* = 15)	12–549/mL (Median 97), 12–322/mL (Median 44)	100% (30/30)	9–29,824 (Median 3109)	Immunofluorescence staining and enumeration, FISH, ICE-COLD PCR, Sanger sequencing, cell culture	[70]
Hydrodynamics (DFF)	Labyrinth (spiral channel w/sharp corners)	7.5 mL	Pancreatic (*n* = 20), M-Breast (*n* = 56)	0–63/mL (Mean 51.6 ± 25.5), 0–21.7/mL (Mean 5.4 ± 4.6)	95% (72/76)	663 ± 647	Immunofluorescence staining and enumeration, single-cell multiplex gene profiling (multiplex qRT-PCR)	[71]
Hydrodynamics (Microvortices)	Vortex HT	~8 mL	M-Breast (*n* = 22), M-Lung (*n* = 15)	0.75–23.25/mL (Mean 5.4), 0.5–24.2/mL (Mean 5.3)	84% (31/37)	187 ± 164	Immunofluorescence staining and enumeration, Single-cell RT-PCR, cell culture, pharmacological studies, single-cell Western blotting	[60]
Dielectrophoresis	Apostream™	7.5 mL	M-NSCLC adenocarcinoma (*n* = 14), Breast (*n* = 20), M-ovarian (*n* = 6), Squamous lung (*n* = 6)	47–216/7.5 mL (Mean 89), 0–36/7.5 mL (Mean 9), 0–5/7.5 mL (Mean 2), 0–4/7.5 mL (Mean 1)	87% (40/46)	--	Immunofluorescence staining and enumeration, phenotypic analysis by laser scanning cytometry	[81]

**Table 4:** Advantages and limitations of biological-based separation methods for CTC isolation.

Separation method	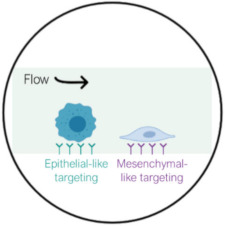 Surface affinity	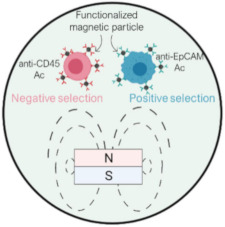 Immunomagnetic
Separation criteria	Surface marker expression of CTCs	Magnetic properties of nano/micro-particles and surface marker expression of WBCs or CTCs
Pros	High sensitivity, specificity, and purity	High sensitivity, specificity, and purity, high throughput, CTC recovery
Cons	Low throughput and challenging downstream analysis	Labeling step is required

**Table 5:** Performances of reported biological-based separation technologies in cell line studies.

Separation Method	Technology	Selection Criteria	Throughput	Sample Composition	Recovery	Viability	Purity	WBC Depletion	Enrichment Factor	Refs.
Surface affinity	^HB^CTC-Chip	EpCAM	1.2 mL/h	PC-3 cells in whole blood	91.8 ± 5.2%	95% ± 0.6%	14.0 ± 0.1%	--	--	[94]
Surface affinity	NanoVelcro Chip	EpCAM	1 mL/h	MCF-7, PC-3, and T24 cells in 1 mL whole blood ^†^	>95%	--	--	--	--	[97]
Surface affinity	NP-^HB^CTC-Chip	EpCAM/Her2/EGFR	1 mL/h	PC3 and MDA-MB-231 cells in 3 mL whole blood ^†^	>90%	Mean 82.5%	Non-specific binding: ~3000 WBCs/3mL	--	--	[99]
Surface affinity	PEDOT NanoVelcro Chip	EpCAM	130 min incubation (capture + release time)	LNCaP, PC3, and 22Rv1 cells in whole blood ^†^	72.5 ± 3.0% ^1^ 75.2 ± 3.2%, 67.8 ± 1.7%	95% after release	46% after release	99.98% after release	4300	[105]
Immunomagnetic	Integrated Ni microstructures	EpCAM	2.5 mL/h	MCF-7, PC3, SK-BR-3, and COLO 205 cells in whole blood	97.3%	--	--	--	--	[110]
Immunomagnetic	MagRC	EpCAM	500 µL/h	MCF-7, SKBR3, PC-3, and MDA-MB-231 in 1 mL whole blood ^†^	93.3% ^2^	98%	--	99.98%	--	[112]
Immunomagnetic	Immunomagnetic nanosphere patterns	EpCAM	60 µL/h	MCF-7 cells, Hep G2 cells, and Cal 27 cells in PBS w/1% hydroxyl propyl methyl cellulose ^†^	~90%	93.1 ± 2.6%	--	--	--	[120]
Immunomagnetic	Spiral channel w/trapping segments and centered magnet	EpCAM	9 mL/h	MCF-7 and MDA-MB-231 cells	96.3 ± 1.5% and 81.2 ± 3.5%	--	--	--	--	[124]
Immunomagnetic	Prism Chip	EpCAM	30 mL/h	PC-3M, LNCaP, VCaP, and 22Rv1 in Hanks’ balanced salt solution w/2% BSA and 5 mM EDTA ^†^	88 ± 6%	91 ± 4%	--	<3 log	--	[125]
Immunomagnetic	μ-MixMACS Chip	CD45 (negative selection)	24 mL/h	MCF-7 cells in whole blood resuspended in 3 mL of PBS with 2% FBS ^†^	90.97%		22.91%	>99%	763.14	[126]
Immunomagnetic	^LP^CTC-iChip (Permeability-enhanced magnetic sorter)	CD45, CD16, CD3, CD45RA, and CD66b (negative selection)	168 mL/h	MGH-BRx-142 cells in 65 mL whole blood ^†,3^	86.1 ± 0.6%	--	0.3%	3.55 ± 0.26 log 99.97%	--	[128]
DFF and Immunomagnetic	Integrated spiral module, passive mixer, and magnetic sorter	Size (15 µm) and EpCAM	3 mL/h to 24 mL/h (8 parallel sorters)	PANC-1 cells in 1 mL whole blood	~90%	--	75%	6 log	--	[133]
DLD and Immunomagnetic	CTC-iChip	Size (3.8 µm) and CD45, CD16 and CD66b (negative selection)	9.6 mL/h	11 different cell lines in 1× PBS with 1% Pluronic-F68 ^†^	98%	--	7.8%	~5 log	--	[135]

^†^ Cancer cells were spiked in low numbers (below 100 cells) to meet clinically relevant cell numbers. ^1^ With the PEDOT NanoVelcro Chip, captured LNCaP cells could be released with 71% efficiency. ^2^ Once the field was removed, 92% of captured cancer cells were recovered from the MagRC device for further offline analysis. ^3^ Cancer cells and WBCs were sorted through a magnetic sorter. RBCs were priorly removed using a size-based inertial separation.

**Table 6:** Performances of reported biological-based separation technologies in clinical studies.

Separation Method	Technology	Blood Sample Volume	Cancer Type	Number of CTCs	Detection Sensitivity	Remaining WBCs/mL	Downstream Analysis	Refs.
Surface affinity	^HB^CTC-Chip	4 mL	M-prostate (*n* = 15) M-pancreatic (*n* = 15)	12–3167/mL (Median 63) 1–57/mL (Median 11)	93% (14/15) --	-- 165–11,190	Immunofluorescence staining and enumeration, molecular characterization (RT-PCR, single-molecule RNA sequencing)	[94,95]
Surface affinity	NanoVelcro Chip	1 mL	Prostate (*n* = 26)	0–33/mL	81% (21/26)	--	Immunofluorescence staining and enumeration	[97]
Surface affinity	NP-^HB^CTC-Chip	3–4 mL	M-breast (*n* = 4)	6–12/mL (Median 7.4)	100% (4/4)	--	Immunofluorescence staining and enumeration, next-generation RNA sequencing	[99]
Surface affinity	PEDOT NanoVelcro Chip	--	Prostate (*n* = 17)	1–7/mL	100% (17/17)	--	Immunofluorescence staining and enumeration, RT-qPCR	[105]
Immunomagnetic	Integrated Ni microstructures	5–10 mL	M-colon (*n* = 1) M-lung (*n* = 1) M-prostate (*n* = 1) M-breast (*n* = 10)	1/5 mL 1/10 mL 13/7.5 mL 0.1–43/mL	100% (13/13)	--	Immunofluorescence staining and enumeration, FISH	[110]
Immunomagnetic	MagRC	10 mL	M-castration-resistant prostate (*n* = 10) Prostate (*n* = 14)	9–48/10 mL 16–95/10 mL	100% (24/24)	2000	Immunofluorescence staining and enumeration, phenotypic profiling	[112]
Immunomagnetic	Immunomagnetic nanosphere patterns	0.6–0.8 mL	M-Lung (*n* = 6) M-Gastric (*n* = 1) M-Gastric antrum (*n* = 1) Lymphatic metastasis (*n* = 1) M-Liver (*n* = 1)	2–12/0.8 mL 6/0.8 mL 9/0.8 mL 4/0.6 mL 9/0.8 mL	100% (10/10)	--	Immunofluorescence staining and enumeration	[120]
DFF and Immunomagnetic	Integrated spiral module, passive mixer, and magnetic sorter	1.4 mL (6.5 mL for miRNA analysis)	Pancreatic (*n* = 14)	14–938/mL (Mean 146 ± 231)	100% (14/14)	0–389 (Mean 42.4 ± 101)	Immunofluorescence staining and enumeration, microRNA and mRNA profiling (qRT-PCR)	[133]
DLD and Immunomagnetic	CTC-iChip	5–10 mL	Melanoma (*n* = 2) Lung (*n* = 9) M-Prostate (*n* = 2) Breast (*n* = 26	1.2/mL 7.9/mL -- 9.6/mL	100% (39/39)	445	Immunofluorescence staining and enumeration, size and phenotypic profiling using imaging flow cytometry	[135]

## Data Availability

Not applicable.
